# Professional training on shared decision making with older adults living with neurocognitive disorders: a mixed-methods implementation study

**DOI:** 10.1186/s12911-020-01197-9

**Published:** 2020-08-12

**Authors:** Moulikatou Adouni Lawani, Luc Côté, Laetitia Coudert, Michèle Morin, Holly O. Witteman, Danielle Caron, Edeltraut Kroger, Philippe Voyer, Charo Rodriguez, France Légaré, Anik M. C. Giguere

**Affiliations:** 1 Department of Family Medicine and Emergency Medicine, Laval University, Pavillon Ferdinand-Vandry, room 2881, 1050 avenue de la Médecine, Quebec, QC G1V 0A6 Canada; 2 Department of Family Medicine and Emergency Medicine, Laval University, Pavillon Ferdinand-Vandry, room 1323, 1050 avenue de la Médecine, Quebec, QC G1V 0A6 Canada; 3 Quebec Excellence Centre on Aging, St-Sacrement Hospital, 1050 chemin Ste-Foy, Quebec, QC G1S 4L8 Canada; 4 Laval University, Pavillon Ferdinand-Vandry, room 4211, 1050 avenue de la Médecine, Quebec, QC G1V 0A6 Canada; 5 VITAM Research Centre on Sustainable Health, Pavillon Landry-Poulin, Door A-1-2, 2nd floor, Room 2416, 2525 Chemin de la Canardière, Québec, QC G1J 0A4 Canada; 6 VITAM Research Centre on Sustainable Health, Pavillon Landry-Poulin, Door A-1-2, 2525 Chemin de la Canardière, Québec, QC G1J 0A4 Canada; 7 Quebec Excellence Centre on Aging, St-Sacrement Hospital, Office L-2, 1050 chemin Ste-Foy, Quebec, QC G1S 4L8 Canada; 8 Pavillon Ferdinand-Vandry, room 3445, 1050 avenue de la Médecine, Quebec, QC G1V 0A6 Canada; 9 Department of Family Medicine, McGill University, 5858 chemin de la Côte-des-Neiges, 3rd floor, Montreal, QC H3S 1Z1 Canada; 10 VITAM Research Centre on Sustainable Health, Pavillon Landry-Poulin, Door A-1-2, 4th floor, Room 4578, 2525 Chemin de la Canardière, Québec, QC G1J 0A4 Canada

**Keywords:** Primary care, Shared decision-making, E-learning, Distance learning, Evidence summary, Decision support technology, Decision boxes, Continued professional development, Aging, Dementia

## Abstract

**Background:**

Shared decision making with older adults living with neurocognitive disorders is challenging for primary healthcare professionals. We studied the implementation of a professional training program featuring an e-learning activity on shared decision making and five Decision Boxes on the care of people with neurocognitive disorders, and measured the program’s effects.

**Methods:**

In this mixed-methods study, we recruited healthcare professionals in family medicine clinics and homecare settings in the Quebec City area (Canada). The professionals signed up for training as a continuing professional development activity and answered an online survey before and after training to assess their knowledge, and intention to adopt shared decision making. We recorded healthcare professionals’ access to each training component, and conducted telephone interviews with a purposeful sample of extreme cases: half had completed training and the other half had not. We performed bivariate analyses with the survey data and a thematic qualitative analysis of the interviews, as per the theory of planned behaviour.

**Results:**

Of the 47 participating healthcare professionals, 31 (66%) completed at least one training component. Several factors restricted participation, including lack of time, training fragmentation into several components, poor adaptation of training to specific professions, and technical/logistical barriers. Ease of access, ease of use, the usefulness of training content and the availability of training credits fostered participation. Training allowed Healthcare professionals to improve their knowledge about risk communication (*p* = 0.02), and their awareness of the options (*P* = 0.011). Professionals’ intention to adopt shared decision making was high before training (mean ± SD = 5.88 ± 0.99, scale from 1 to 7, with 7 high) and remained high thereafter (5.94 ± 0.9).

**Conclusions:**

The results of this study will allow modifying the training program to improve participation rates and, ultimately, uptake of meaningful shared decision making with patients living with neurocognitive disorders.

## Background

The care of older adults living with neurocognitive disorders (NCDs) requires making difficult decisions. For instance, the disabling and multi-morbid nature of this condition involves selecting services to reorganize daily life, choosing pharmaceutical or non-pharmaceutical treatments, and preparing advanced care plans and directives. Because there are generally several acceptable options for these decisions, decision making should consider the experiences, preferences, and values of the older adult living with NCDs and their family or friend caregiver. The shared decision-making (SDM) process is ideal for guiding decision making in this context, as it relies on a discussion among all parties to balance evidence-based healthcare information, the expertise of the healthcare professional (HCP), and the experiential knowledge, values, and preferences of the person living with NCDs and their family/friend caregivers. A large systematic review recently established that SDM helps improve patients’ knowledge of the options, congruency between their values and care choice, comfort with the decision, and engagement in decision making [1]; however adoption of SDM by HCPs in their routine practice is still suboptimal [2].

SDM implementation in the context of caring for older adults with NCDs remains largely unexplored. Implementation studies have been conducted in nursing home residents living with NCDs [3, 4], and a single study has been completed to date to implement SDM among the interprofessional care team, family/friend caregivers, and community-based older adults living with NCDs, for housing decisions [5]. Decision making in the context of NCDs is particularly challenging, as decisions are often emotionally laden [6] and complicated by the disease, ethical and legal dilemmas, and the presence of multiple stakeholders [7]. As a result, older adults living with NCDs are typically excluded prematurely from decision making [7]. Hence, there is a need for studies to inform the implementation of professional training and patient/caregiver decision aids to support SDM among community-based older adults living with NCDs, their caregivers, and interprofessional teams.

A recent systematic review of 15 studies highlights a lack of evidence on the effectiveness of different types of interventions to improve SDM adoption among HCPs, such as educational meetings, educational material, educational outreach visits, and reminders [8]. Furthermore, although an environmental scan described 168 validated professional training programs in SDM [9], the assessment of their effects and implementation is heterogeneous, and there is still a lack of evidence on the best practices to develop, implement, and assess these training programs [10]. Participation in continuing professional development (CPD) strategies is challenging for HCPs, especially for those who work in remote areas and need to travel long distances to take part [11, 12]. A recent systematic review suggests that remote online training, or e-learning, could be more accessible—and equally effective—as face-to-face training [13].

We thus set out to study the factors influencing participation in a professional e-learning program on SDM comprising an e-learning activity and five Decision Boxes on the care of older adults with NCDs, by addressing three specific questions:

1. What is the level of participation of HCPs in the various components of the program?
2. Which factors influence HCPs participation in the program?
3. What are the effects of the program on HCPs’ knowledge about and intention to adopt SDM with these patients?

## Methods

### Description of the training program

This professional training program included (1) a self-directed e-learning activity on SDM, lasting about 1 h, that participants could complete in several sittings at their work location or at home; and (2) five evidence summaries, or Decision Boxes (DBs), to support decision making at the point of care.

The generic e-learning activity included four successive training modules that aimed to 1) explain SDM and its implementation in daily practice; 2) describe strategies for determining patients’ values and preferences; 3) describe strategies for communicating probabilities to patients; and 4) explain how to incorporate SDM into clinical encounters with patients. The minimum duration of each module was respectively 9 min for Module #1; 20 min for Module #2; 6 min for Module #3; and 28 min for Module #4. The design of this activity was based on our team’s earlier work on CPD training to support SDM for acute respiratory tract infections [14].

The series of five evidence summaries described the options available to older adults living with NCDs who are faced with five important and frequent decisions that we identified in an earlier study (Table 1) [15]. We designed these evidence summaries as Decision Boxes (DBs), which are meant to provide stakeholders with evidence in a format supportive of SDM (i.e., one that avoids biasing decision making by concisely setting out the pros and cons of all available options, in absolute risks) [16, 17].

**Table 1:** Decision Box titles and level of participants’ interest for each title

Distribution order	Decision Box title	Interest in the title, on a scale from 0 to 100 with 100 high Mean (SD); (*n* = 47)
1	Choosing a non-pharmacological treatment to manage agitation, aggression, or psychotic symptoms	87 (11)
2	Choosing an option to maintain quality of life	85 (13)
3	Choosing a support option to decrease caregiver burden	80 (18)
4	Deciding whether or not to stop driving following diagnosis	78 (30)
5	Deciding whether or not to prepare a power of attorney for personal care^a^	67 (32)

^a^Power of attorney for personal care is known as a “protection mandate” in Quebec, Canada, and covers health, property, and financial matters

Professionals who completed training (arbitrarily defined as completing the four e-learning modules and any one of the five DBs), were entitled to training credits. Participation was otherwise voluntary, since we offered no other incentives.

In parallel with this professional training program, we also designed and evaluated patient decision aids for each of these decisions, a project that is reported elsewhere [18].

### Study design

**Fig. 1:**
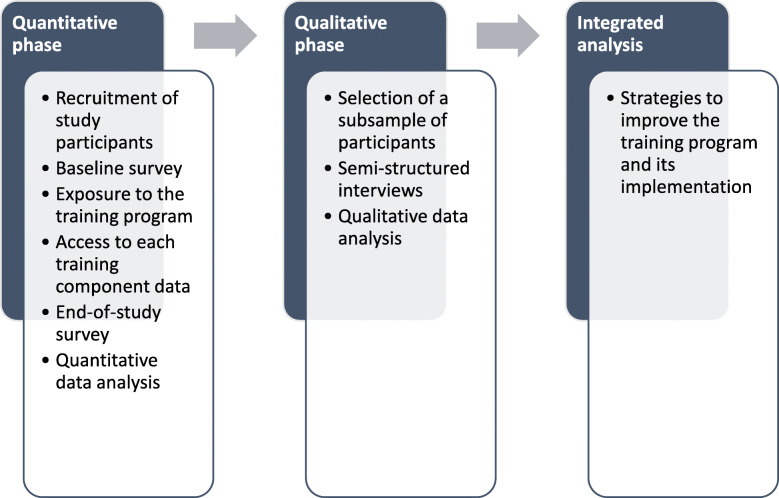
Study design comprising a description of the quantitative and qualitative data collections

In this explanatory, sequential, mixed-method study, we assessed participation in each training component using quantitative access data (Fig. 1). We also asked participants to complete quantitative questionnaires before and after training, to assess the effects of the training program on their knowledge and intention. Our hypothesis was participants’ knowledge and intention would increase after training. Then, we sought to understand the factors influencing participation using semi-structured individual interviews with a subset of participants selected based on the results of the quantitative phase.

We originally planned this study as a clustered randomized trial that is described elsewhere [19]. In short, this study aimed to assess the effectiveness of the professional training program in increasing the empowerment of older adults living with NCDs and their caregivers to make health-related decisions. Unfortunately, we experienced low patient recruitment rates and rather studied the implementation of this training program and its effectiveness in influencing variables at the HCP level.

### Participants

We recruited a convenience sample HCPs from various professions (e.g., family physicians, nurses, and social workers) who practiced in family medicine clinics and homecare services in the province of Quebec, Canada. We presented the study to the HCPs during one of their regularly scheduled team meetings. Those who agreed to participate signed an informed consent form, completed a study entry questionnaire on their sociodemographic and professional characteristics (age, gender, profession, years of practice, city size), and responded to a question to assess their interest in each DB topic, on a slider scale ranging from 0 to 100, with 100 high.

### Implementation strategy

The participants received an email with the access codes to the e-learning activity, and to the DB. After this first email, we sent them four more emails, one every 2 weeks, to give them access to each DB and to remind them of the e-learning activity. Overall, the participants had access to the training program from February 2018 to May 2018. The DBs were distributed in the same order to all participants, starting with the topic that they rated as the most interesting, on average, and then in decreasing order of interest (Table 1).

### Quantitative data collection

#### Survey

The study participants completed an online survey before and after the e-learning activity. The survey included 27 questions: (A) one question on their prior training in SDM; (B) one question inspired by the Ottawa Decision Support Framework to assess their knowledge about SDM [20]; (C) one question to assess their knowledge about risk communication; (D) two questions with case base scenarios for each DB topic, to assess participants’ perceived awareness of the available options and awareness of the options; (E) eight questions, including case-based scenarios, to assess clinical knowledge relative to the care of older adults living with NCDs; (F) five questions to evaluate participants’ intention to use SDM with their next patient facing a preference-sensitive decision, and the determinants of this intention (attitude, beliefs about capabilities, moral norm, and social influence), using a brief 5-item version of the CPD-REACTION [21]; (G) eight questions to assess perceptions of their ability to adopt SDM using the novel IcanSDM scale [22]; and (H) one question to assess their preferred role in decision-making [23].

#### Access data

The website that supported e-learning activity allowed recording participants’ access to each training component, as well as the time spent on each module in the e-learning activity. We were also able to record participants’ access to the DBs when they answered a questionnaire about their experience using the tool, the results of which are reported elsewhere [24].

### Qualitative data collection

#### Selection of a participant subsample

We aimed to recruit a subsample of 16 people from among the participants, to interview them about the factors that encouraged or restricted their participation in training. We estimated that this sample size should allow saturation since the sample was relatively homogenous and the questions discussed were straightforward and practical. Among the 16 people, we planned to recruit eight who had fully participated in training and eight who had not. To recruit people who had participated in training, we had a question in the survey that they completed after training, asking their permission to contact them by phone for a 30-min individual interview. We recruited people who had not participated by email.

#### Procedure

We conducted the individual phone interviews (roughly 30 min in length) 1 month after participants had completed the professional training program. We used a semi-structured interview guide to elicit (1) their attitude towards the training program, and (2) their beliefs about their capabilities to complete the training program. The interview guide was based on the Theory of Planned Behaviour [25], according to which a behaviour may be predicted by a person’s intention (motivation) to adopt it, and a person’s intention may, in turn, be predicted by several determinants, including belief about consequences, social influence, and beliefs about capabilities. We also asked a few questions to explore how they used what they learned through the program in their encounters with patients.

We recorded the interviews using audio-digital recorders, and transcribed the discussions verbatim.

### Analyses

We completed descriptive analyses of all quantitative data. We used simple logistic regressions to identify the factors influencing completion of the training program. To this end, we first performed univariate between completion and each of the potential factors (sociodemographic and professional characteristics, prior training in SDM, interest for each of the DB topic). We then tested a simple logistic regression model comprising all the factors that demonstrated a significant effect on completion. All quantitative statistical analyses were conducted using the SAS statistical package (SAS Institute Inc), and bilateral statistical tests were performed at a significance level of 0.05.

We also performed bivariate analyses to compare questionnaire responses before and after the training program. In addition, we used student-dependent paired T-tests to compare mean scores and the Fisher test to compare proportions before and after training.

For qualitative data, three researchers independently conducted a thematic content analysis using an deductive approach, initially based on the individual factors described by the Theory of Planned Behaviour [25], then on the emerging themes of the discussions. The analyses aimed to describe the factors encouraging and restricting participation in the training program.

We then explored the qualitative results to discover any confirmation, contradiction, cross-validation, or corroboration of the quantitative results [26]. Drawing on the various sources, the research team met to suggest strategies to improve the training program and its implementation.

We obtained ethical approval for this project from the ethics review board of the *Ministre de la santé et des Services Sociaux* (reference CCER15–16-05) and the *Centre Hospitalier Universitaire de Québec* (reference 2016–2521). All participants signed consent forms for the study.

## Results

### Participants

**Fig. 2:**
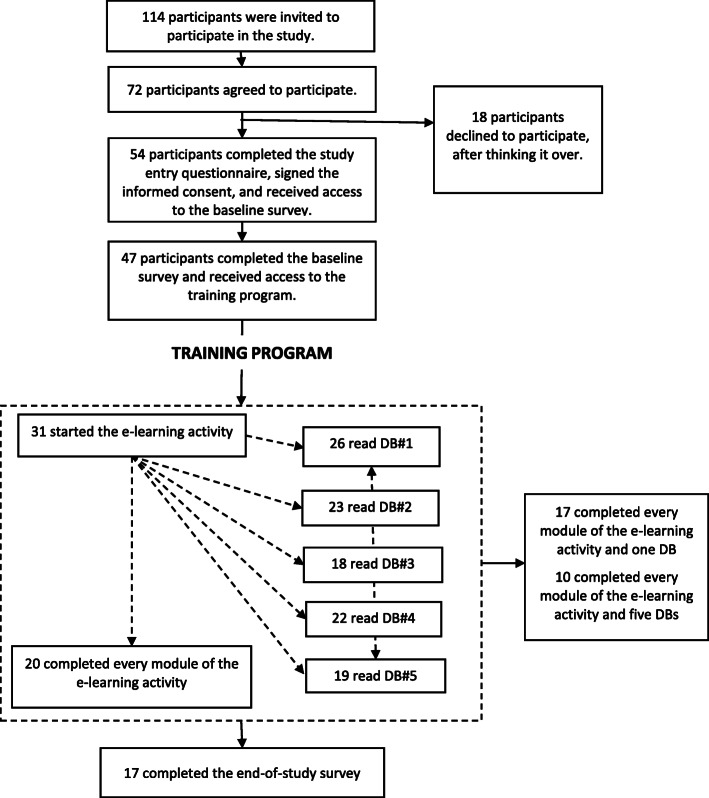
Flow of participants (see Table 1 for DB titles)

Of the 114 HCPs invited to participate, 72 (63%) accepted (Fig. 2). Eighteen left the study before signing the informed consent and completing the study entry questionnaire. Of the 54 participants who signed the informed consent and completed the study entry questionnaire, 47 completed the baseline survey and received access to training. Of these 47 people, 17 (36%) completed the final survey after training.

Most of the 47 participants who completed the baseline survey were women (83%) (Table 2). They represented several professions, but most were physicians (36%), nurses (21%), or social workers (21%). Most of them (81%) had never had any training in SDM. They reported a mean interest in the topics covered in the DBs of 80% (± SD 14%); they were most interested in DB#1 (Non-pharmacological treatment to manage agitation, aggression, or psychotic symptoms) (mean interest = 87 ± SD 11%) and least interested in DB#5 (Deciding whether or not to prepare a power of attorney for personal care) (mean interest = 67 ± SD 32%) (Table 1).

**Table 2:** Characteristics of study participants

Participant Characteristics	Frequency (Total ***N*** = 47)	
	n	%
**Age (years)** ^**a**^		
Under 30	7	15.2
30–39	15	32.6
40–49	15	32.6
50–59	8	17.4
60–69	1	2.2
**Gender**		
Female	39	83.0
Male	8	17.0
**Profession**		
Physician	17	36.2
Nurse	10	21.3
Social worker	10	21.3
Occupational therapist	6	12.8
Nursing assistant	1	2.1
Dietician	1	2.1
Pharmacist	1	2.1
Physiotherapist	1	2.1
**City size where practice located**^**b**^		
Rural area	0	0.0
Small city	6	12.8
Mid-size city	0	0.00
Large city	41	87.2
**Years of practice**^**c**^		
< 10	18	38.3
10–19	11	23.4
20–29	12	25.5
30–39	3	6.4
Do not recall	3	6.4
**Received prior SDM training**		
Yes	9	19.2
No	38	80.9

^a^One missing data for age

^b^*City size < 1000 = rural; 1000–29,999 = small; 30,000–99,99 = mid-size; > 100,000 = large* (Statistics Canada, 2011)

^c^*Years of practice = year of data collection (2018) – year practice license was granted*

### Level of participation in the training program

Of the 47 participants who completed the baseline survey, 17 (36%) completed the four modules of the e-learning activity in addition to reviewing a minimum of one DB; 10 (21%) completed the four e-learning modules and reviewed five DBs (Fig. 2). If we consider the DBs exclusively, 26 of the 47 participants (55%) reviewed at least one DB.

Completion time of the entire e-learning activity ranged from 40 min to 9 h, for an average duration of 57 min. Some participants spent as long as 5 h on the introduction, while 68% of the 47 participants spent less than 30 min on it. These estimates could, however, reflect the time during which people were connected to the activity without being actively engaged in doing the training.

Of the 20 participants who completed all the modules of the e-learning activity, four took 2 h30 to complete it (20%), and 16 took less than 2 h (from 47 min to 1 h52). The average time to complete the modules 1, 2, 3, and 4 were respectively, 29 min (±SD of 23 min; range 5–95 min), 38 min (± 68 min; range of 4–255 min), 6 min (±4 min; range of 2–19 min), and 17 min (±10 min; range of 6–44 min).

Of the 11 participants who accessed the e-learning activity and did not complete all the modules, nine stopped after less than 5 min on the Introduction or on Module 1 (82%), and two completed the first two modules only, in about 1 h (18%).

The logistic regression to describe the factors influencing participation in the training program gave no statistically significant factor explaining completion.

### Interview findings: factors influencing participation in the training program

We recruited and interviewed 11 participants instead of the 16 planned, since we reached data saturation in the last interviews conducted when we failed to record any new emerging theme [27]. Of these 11 participants, six had completed all the modules of the e-learning activity and one DB, and five had not.

These interviews allowed us to identify several factors encouraging or restricting participation in training, with regard to participant attitudes and beliefs about their capabilities. The study of these factors then led us to pinpoint specific strategies for improving the training program and its implementation. These findings are described in the next paragraphs.

#### Factors encouraging participation

Both the participants who fully completed the training program, and those who partially completed it, had general positive attitudes towards it (Table 3). They perceived the training program as useful for learning about SDM, for improving their management of the problems faced by older adults living with NCDs, and for improving their communication with them. Participants especially appreciated the fact that training allowed them to become aware of the DBs and other patient decision aids. Some participants mentioned that the DBs covered topics of interest for practice, and that they helped meet their clinical needs. Several participants also pointed to the usefulness of the DBs because they presented various interventions, with their pros and cons. A number of participants mentioned how completing the program trained them to communicate understandable information on all the options to patients, and to provide them guidance.

**Table 3:** Factors encouraging participation in the e-learning program

Factor	Sample citation (source)^a^
Attitude	
**The program is useful for learning about SDM**	“For me it was an introduction to the concept of shared decision making, it raised my awareness about it.” (Occupational therapist #29, home support service #3)
**The program is useful for practice**	
Provides ideas on ways to manage NCDs problems	“It has all kinds of information sources that are useful for my practice. I’ve had a slew of interventions in BPSD (behavioural and psychological symptoms of dementia) recently, and it was a good source of inspiration.” (Occupational therapist #29, home support service #3) “I thought the Decision Boxes were fun. They helped give us ideas on how to conduct interviews using shared decision making. Even though there are some topics that don’t have Decision Boxes, they were still useful tools for understanding how to interact with patients.” (Dietician #9, home support service #1)
Provides an introduction to DBs	“It was interesting to learn that decision aids exist. And they were well made. The training was well organized, and it included information on the Laval University and Ottawa sites where you can find them. It was good to know.” (Dietician #9, home support service #1)
DBs cover topics of interest for practice	“I found the topics interesting and felt they could be very useful in my practice.” (Physician #55, Clinic #1)
DBs help meet clinical needs	“The training informed me of alternative non-pharmacological treatments to meet the needs of clients.” (Nurse #65, Clinic #1)
DBs present various interventions and their pros and cons	“It helps us move beyond the scope where the nurse has the answer, since it’s the client, instead, who has to choose. But at the same time, to make a choice, they need to understand the pros and cons, so they can make an informed decision. In some cases, it’s not as easy as that, but with these Decision Boxes, it really gives us a good idea of what the options are, as well as the pros and cons, in pretty simple terms. It’s good.” (Nurse #26, Clinic #4)
DBs facilitate the communication of information to patients	“It’s something I consider necessary, that is, being able to provide information that is easily understood by our clientele, by the patient, and their loved ones. The training program gives us an appreciation of the work that’s been done to help facilitate our task of delivering information in a format that’s easy to understand, and that can be consulted by the patient’s family not during the encounter, but afterwards.” (Physician #73, Clinic #2) “The training gives us a good understanding of how to guide patients using evidence, according to the topic, like driving or how to provide support to caregivers—things like that. I think it helps us see all the possible avenues, with their pros and cons, and it helps us provide guidance to the people we work with.”(Physiotherapist #46, home support service #2)
Beliefs about capabilities	
**Ease of access**	
DBs are printable	“I plan to print out the Decision Boxes in question. I posted them on the family medicine intranet because I, personally, find them very useful. Once I have access to a colour printer, I plan to collect them together in a binder, so I can access them in the clinic and use them in a teaching context with our resident doctors so that they, too, can use them in their interactions with patients. Plus, I’ll make available the Decision Boxes designed for patients and/or their loved ones.” (Physician #73, Clinic #2)
Value of having one version of the DBs for clinicians and a simpler version for patients	“What’s interesting too is that there’s a part that’s really more for the professional, to guide their intervention, and a simpler part that’s more for the patient.” (Social worker #20, home support service #1)
Access to DB information in practice is quick due to their brevity, standardized presentation of information, and separate DB for each clinical situation	“What I found interesting with the modules is that you can seek out certain specific parts. For example, if I’m faced with Problem X, I can go straight to the Decision Box on that particular topic. It’s easier than having to wade through a long module that’s not divided into topics, and where you have to search to find your information. But with the short Decision Boxes, you can quickly find what you’re looking for.” (Social worker #20, home support service #1)
DBs are available to patients/caregivers after the consultation if they require more information	“What is also interesting is that the DBs are available in a format that can be consulted after the consultation by patients and their families.” (Physician #73, Clinic #2)
Short modules make it easier to retrieve information from the e-learning activity	“The training module is interesting too. It’s concise, not too long, and the sheets are pretty quick to complete. I think it’s a winning formula. The fact that it’s short and concise makes it easier to use.” (Nurse #26, Clinic #4) “They’re really easy to use and to find your way around.” (Nurse #65, Clinic #1)
The training program is easy to do: brief, concise, clear, well-explained	“I liked the fact that it’s not too long, it’s set out clearly, it’s well explained. It was quick to use.”(Nurse #65, Clinic #1)
Flexible nature of the training program: easy to access at the most convenient time for the learner, and at their own pace	“It’s good that it is possible to access it at the moment we choose, at the right moment: it’s the flexibility.” (Physician #73, Clinic #2)
**Ease of use**	
No prerequisite for the training program	“There are not really any prerequisites; I would say that anyone working in a clinic with a minimum level of experience would be able to complete it.” (Nurse #26, Clinic #4)
Training program provides easy-to-understand, visual and practical training	“I found it visually appealing, and the fact there were examples gave me a better ideas on how to interact with my patients.” (Dietician #9, home support service #1)
Applicability of learnings to other clinical situations	“I think that it could be used afterwards for other types of clienteles. The Decision Boxes incorporate a slightly more standardized practice when it comes to sharing information with the clientele, and to shared decision making.” (Nurse #26, Clinic #4)
DBs are well explained and provide concrete guidance	“The DBs are relatively short (2–3 pages), there’s not too much information. They’re easy to find your way around, easy to follow and to use in the workplace.” (Social worker #20, home support service #1)
**Extrinsic sources of motivation**	
Incentives: continuing education credits	“There’s an incentive with the training units.” (Occupational therapist #29, home support service #3)
Participation encouraged by reminders and follow-ups during training	“I thought the emails you sent to remind us and to inform us when new Decision Boxes were available was a good approach.” (Dietician #9, home support service #1)

^a^Citations were translated from French

The participants reported several factors encouraging their participation with regard to their beliefs about their capabilities to participate. Most mentioned ease of access to the training program as a factor encouraging their participation. They mentioned that it was easy to do, as it was concise and clear, and they appreciated the short modules of the e-learning activity that made it easier to retrieve information. They also valued the DBs from a practical point of view, noting that accessing the information in the DBs is quick due to their brevity, the standardized presentation of information, and the availability of different DBs for each clinical situation. They further appreciated that DBs were printable, and mentioned their convenience as a source of information for patients/caregivers after the consultation. Participants also appreciated that completing training did not require any prerequisites. They also found that the training program was easy-to-understand and visually appealing, and they appreciated that it provided practical training. The participants reported that they found the DB well explained and that they offered practical guidance. One participant also mentioned how learning was applicable to other clinical situations. Participants also mentioned that extrinsic sources encouraged their participation, in particular the email follow-ups and the associated continuing education credits.

The participants made several suggestions to improve participation to the training program, the most important of which was to shorten training and integrate it formally into participants’ working schedules. Some people also mentioned that it would be desirable to be able to adjust the speed of the narration. Regarding implementation, the participants suggested extending dissemination of the DBs, especially to employers and decision-makers.

#### Factors restricting participation in the training program

All of the factors mentioned by participants as limiting their participation were aspects of their beliefs about their capabilities to participate (Table 4). The factor most frequently reported was their lack of time, or the time required. Participants explained how it was generally difficult for them to find time to complete training. They also mentioned that time required to adopt the tools may be longer for professionals lacking experience in the topic. A number mentioned that the period selected to distribute the program was suboptimal because it was a particularly busy time.

**Table 4:** Factors restricting participation in the e-learning program

Factor	Sample citation (source)^a^
Lack of time / time required	
Poor match between the duration of the training and learners’ time constraints	“Time is the real sticker these days. It’s very difficult in today’s home support system. We’re overloaded, we have interviews with patients to conduct using CDSSs (computerized clinical decision support systems) that last hours, we’re under enormous pressure, and we don’t have time to stop.” (Occupational therapist #45, home support service #2)
Unfavorable period (note from author: training ran from February to May 2018)	“With the whirlwind of life, with the daily routine of my tasks as a family doctor, to do this in excess, at midnight or 1 am, it is not possible, at least not at that time, with all my tasks.” (Physician #55, Clinic #1)
No time slot dedicated to the training activities	“If you look at our work situation, of course it’s not always easy to take the time to stop and do it. The barriers we face are that we have many other tasks to do. I’m too busy to start wearing several hats at a time, so the barriers aren’t the training itself; it’s the fact it takes time, and that you have to make that time.” (Physician #73, Clinic #2)
Prioritization of clinical, family, or administrative activities limiting the time available for training	“I often work until midnight, and it’s not for additional training … So, training comes after all the other priorities.” (Physician #55, Clinic #1)
Time required to take ownership of the tool may be longer for those professionals lacking experience in the topic	“Of course, taking ownership of the tool also takes time.” (Physiotherapist #46, home support service #2)
Inconvenience of training components	
Training fragmented into e-learning activity and evidence summaries makes it difficult to follow; multiple stages	“I think I would have preferred to do it all at once.” (Social worker #50, home support service #2)
DB content not well adapted to some professional fields of practice (e.g., dieticians)	“The Decision Boxes are certainly interesting, but they don’t apply to every situation. There weren’t any in my area of practice. So for me, it’s a bit less motivating. I don’t think I’ll be using any of the Decision Boxes that have been mentioned to date.” (Dietician #9, home support service #1)
Lack of information on how to use the DBs	“I gave all the information verbally. I didn’t know you could hand the DBs over to the patients. I only used the DBs designed for the clinicians and I didn’t have the reflex to use those designed for the patients.” (Nurse #26, Clinic #4)
Technical / logistical barriers	
The audio can be a disturbance for co-workers in a shared office	“We share our offices. I don’t have headphones, so the training has to be done at precise times, so as not to disturb my colleagues. It could be harder to find the right time, but it’s really just a very minor point.” (Occupational therapist #29, home support service #3)
Issues finding where the learner left off in the event of pausing	“I thought that when we paused the training and came back to it later, we could just pick up where we left off. But that’s not what happened. Every time I came back to it, I had to enter the exact place I’d left off and keep clicking on “Forward” until I got back to the right spot. So that was a bit complicated, because sometimes I couldn’t remember where I’d got to.” (Dietician #9, home support service #1)
Issues with Internet	“As far as I’m concerned, I have a lot of trouble with things on the Internet. I’m the type to be more face-to-face.” (Dietician #9, home support service #1)
DB not optimized for viewing on smartphones	“I don’t have a computer at home, so I did it on my cellphone, and it wasn’t easy to do.” (Social worker #50, home support service #2)
Social barriers	
No discussion to reflect on lessons with peers	“In my workplace, I was the only one doing it. It would have been good to have colleagues doing it at the same time so we could discuss it.” (Occupational therapist #29, home support service #3)
Negative influence of colleagues who did not complete the training program	“I suspect I’m not the only one at the clinic who didn’t have time to complete the online training because we have a lot of reading to do, forms to fill out, a family life when we can, so adding this on top.” (Physician #55, Clinic #1)
Lack of formal recognition of training by the employer	“It would have been good to receive more official recognition from the employer.” (Physiotherapist #46, home support service #2)
Evidence summaries unknown to/not popular with colleagues	“There is a lack of awareness that there are decision-making tools. They should be promoted even more.” (Physiotherapist #46, home support service #2)
Difficulty in using DBs	
Costs associated with printing the DBs	“The printouts, especially the colour ones, which are more attractive, can generate costs, especially if you need them for the patients and their families as well.” (Physician #73, Clinic #2)
Preparation required before using them during consultation (access, printing, etc.)	“It takes a lot of steps to go find the link to access the tool, to go on the internet, to then be able to print it. It is a good tool, but it would be good to have it at your fingertips so it can be used. And since I didn’t have it at my fingertips ...” (Physician #73, Clinic #2)
Some figures in the DBs difficult to interpret and less relevant	“There are, for example, times when the percentages are not easy to interpret or apply. There are some that are relatively easy, like indoor gardening. As advantages, we see that agitation decreases for 64% of seniors: it is relatively easy. But for others, it is sometimes less obvious. We understand that the therapeutic touch decreases restlessness in 28–54% of cases. We understand that it can reduce agitation, but I don’t think I will use these figures, I will use the averages more.” (Social worker #20, home support service #1)
DBs not available for all patients	“When we’re at the clinic with patients, there are often a number of priorities to be addressed. It’s rare that there’s only one reason for consulting, and that that reason happens to be one of the ones addressed in the Decision Boxes.” (Physician #73, Clinic #2)
Other tools already handed out to patients; DBs not yet incorporated into regular practice and can be cumbersome	“We give out lots of advice and we hand out all kinds of stuff, plenty of documents. We haven’t gotten around to giving out additional tools. It just hasn’t been part of our practice so far.” (Occupational therapist #45, home support service#2)

^a^Citations were translated from French

Among the other factors limiting participation, we identified certain disadvantages of the training components themselves, technical or logistical barriers, social barriers, and difficulties in using DBs. For example, some participants reported that spacing out the training elements for several weeks made the training more difficult to follow. The technical barriers reported consisted in difficulties accessing the Internet access or navigating the website. Participants also indicated that their colleagues and employer influenced their determination to complete the training. Lastly, participants indicated that the use of DBs entailed certain costs and required adapting them to each patient individually before integrating them formally into their practice.

#### Strategies for improving the training program and its implementation

Based on the factors identified that encouraged and restricted participation in training, on the strategies proposed by participants themselves, on authors’ experience and on scientific evidence, we identified a set of strategies for improving the learning components used in the training program, as well as strategies to improve its implementation (Table 5). A few of the proposed strategies address time constraints, such as officially incorporating training into the participant’s schedule and adapting training length to individual needs and experience. Other proposed strategies address the inconvenience of the training components, such as facilitating access to the DBs, making the online activity available in print format for regions with limited Internet access, and including a user’s guide for those learners who are less Internet-savvy.

**Table 5:** Strategies to improve training program on shared decision making with older adults with neurocognitive disorders

Strategy	
To improve learning modalities	
Offer the option of receiving the DBs in a single block rather than in sections.	
Make the online activity available in print format for regions with limited Internet access.	
Subdivide the longer modules.	
Use podcasts.	
Give participants the option of skipping the modules on topics they are already familiar with.	
Clarify the availability of the tools throughout the training program, and promote their potential as a teaching aid for interns.	
Create the possibility for learners to adjust the speed of the narration in the videos.	
Make headphones available to learners in shared workspaces.	
Make it easier to pick up training again after pausing.	
Include a user guide for learners who are less tech-savvy.	
To improve implementation	
Include targeted messages to help promote the training program:	
- By clarifying learners’ preferred objectives (understanding SDM, learning about the tools, understanding the evidence about the different interventions)	
- By highlighting the clinical issues covered by the DBs, since they are practice-oriented	
- By promoting the usefulness of DBs to communicate information to patients	
Maintain training credits as a source of motivation, enhance them if possible, and add other possible sources of motivation.	
Make the training program shorter.	
Officially incorporate the training into the participant’s schedule by negotiating with immediate superior.	
Provide training at a more convenient time of the year, e.g., in summer.	
Adapt training length to individual needs and experience.	
Make DBs easier to access:	
- Facilitate patients’ access to online DBs, e.g., by giving them the website address	
- Create direct access links to the DBs in the EMR (Electronic Medical Record)	
- Create direct access links to the DBs and the e-learning activity directly on clinic websites	
- Offer colour printed versions (budget for them) or equip offices with colour printers	
Incorporate short modules specific to each clinical intervention field.	
Create DBs for all of the themes addressed in clinical encounters, and expand the practice areas covered.	
Offer learners the chance to choose the DBs they wish to review, at the beginning of the training program.	
In the online activity, present examples, clinical cases, or role-plays relating to various scopes of professional practice.	
Simplify the data presented in the DBs.	
In the online activity, explain how to present the wide confidence intervals associated with effect estimates.	
Promote the tools with decision makers and employers (nursing or multidisciplinary department heads, professional bodies, universities), via webinar, for example.	
Address the barriers mentioned during the learning program with presentations and credited workshops, in collaboration with officially recognized public authorities.	
To improve dissemination of the tools, make them available in clinics, health institutes, libraries, and other public places.	
Promote the option of doing the training as a group.	
Offer incentives to participate, in the form of gifts, money, or meals.	
Promote shared decision making in the population and directly support patients and their caregivers in participating to the clinical decision making process.	
Promote shared decision making at level of the government.	

### Survey results: effects of the training program

We partially confirmed our hypothesis to the effect that participants’ knowledge and intention would increase between before and after training.

We did not observe any change in participants’ knowledge about SDM after training (Table 6). By contrast, knowledge about risk communication statistically improved (*P* = 0.02). Moreover, we observed statistically significant improvements in HCPs’ awareness (*P* < 0.001) and perceived awareness (*P* < 0.01) of the options after training. Training had variable effects on clinical knowledge, depending on the topic. Participants’ level of intention to adopt SDM did not change between before and after training. Intention to adopt SDM was high at baseline, and remained high after training (mean before = 5.88 ± 0.99; after = 5.94 ± 0.90, on a scale ranging from 1 to 7, with 7 the highest intention).

**Table 6:** Effects of the training program among participants who completed the survey before and after training

Outcome		Before training	After training	*P*-value
Knowledge				
Knowledge about SDM	Mean score (SD) (scale 1–5, with 5 high)	4.2 (1.33)	3.9 (1,45)	0.31
Knowledge about risk communication	Number of people with correct answers, n (%)	5 (29.4%)	9 (52.9%)	0.02
Perceived awareness of the options (3/5 Decision Boxes)	Mean score (SD) (scale 1–5, with 5 high)	3.0 (0.78)	3.9 (0.56)	0.0006
Awareness of the options (3/5 Decision Boxes)	Mean proportion of correct answers (SD)	16.7% (10.9%)	42.2% (32.6%)	0.0011
Clinical knowledge				
*Deprescribing antipsychotics*	Number of people with correct answers, n (%)	0 (0.0%)	4 (23.5%)	0.78
*Impacts of stopping driving*	Mean (SD) (scale 0–5, with 5 high)	2.0 (1.2)	4.1 (1.3)	0.0004
*Strategies to communicate about stopping driving*	Mean proportion of correct answers (SD)	20.0% (18.7%)	25.9% (22.1%)	0.07
*Risk factors for caregiver burden*	Mean (SD) (scale 0–4, with 4 high)	3.0 (1.0)	3.2 (1.1)	> 1.000
*Awareness of the information to provide patients to reflect on the Power of attorney*	Mean (SD) (scale 0–4, with 4 high)	2.7 (1.1)	2.6 (0.8)	0.72
*Characteristics of the power of attorney*	Mean (SD) (scale 0–4, with 4 high)	2.6 (1.1)	3.3 (0.6)	0.082
*Elements to check before recommending a treatment to a vulnerable senior*	Mean proportion of correct answers (SD)	32.4% (30.3%)	35.3% (29.4%)	0.79
Intention to adopt SDM	Mean score (SD) (scale 1–7, with 7 indicating high intention)	5.88 (0.99)	5.94 (0.90)	0.83
Perceived ability to adopt SDM (IcanSDM)	Mean score (SD) (scale 1–10, with 10 indicating high ability)	6.54 (1.58)	6.85 (1.25)	0.43
Role preference	Number of participants n (%)			
I make the decision alone, relying on the best scientific evidence available		0 (0.0%)	0 (0.0%)	0.82
I make the decision, but strongly considering the opinion of the patient		0 (00.0%)	3 (17.7%)	
The patient and I make the decision together equally		4 (23.5%)	4 (23.5%)	
The patient makes the decision, but strongly considering my opinion		5 (29.4%)	2 (11.8%)	
The patient makes the decision alone, after obtaining information on the best available scientific evidence		8 (47.06)	8 (47.1%)	
Self-reported use of the training material to answer questions after training				
Yes	Proportion n (%)	NA	9 (53)	NA
No		NA	6 (35)	NA
No answer		NA	2 (12)	NA

None of the determinants of intention measured through the CPD-REACTION (beliefs about capabilities, beliefs about consequences, social influence, moral norm) changed between before and after training. There was either no significant difference in participants’ perceived ability to adopt SDM and in their preferred role in SDM before and after the training program. About half (53%) the participants who completed the questionnaire after training reported having consulted the training material again after training to answer questions that came up.

The trends regarding these different variables before training were similar between participants who fully completed the training and those who partially completed the training.

### Integration of findings

The interviews highlighted certain factors that help understand the low participation rates in training, and high attrition during training, such as lacking time and social support, technical and logistical barriers, and difficulties using the DBs. Although our quantitative results suggest that knowledge about SDM did not change between before and after, we observed positive attitudes towards SDM in participants who had completed it. We also note that, during the interviews, participants reported using the training material to answer questions after the training was over, and printing out and sharing the DBs with their colleagues and the residents under their supervision. Some participants also reported that they intended to integrate the DBs into their clinical and teaching practices. These findings concur with the high levels of intention to adopt SDM that we measured before and after training.

Additionally, our findings from the interviews about participants’ appreciation of the usefulness, ease of access, and use of the training program converge with our quantitative results demonstrating an improvement in participants’ clinical knowledge after training.

## Discussion

In this mixed-method study, we described the level of participation of HCPs in a multi-component training program on SDM in the context of NCDs, and the factors influencing their participation. We found that, among those who had initially agreed to participate, only 24% (17/72) completed the training program. Qualitative interviews with HCPs revealed several factors restricting their participation, such as their lack of time to complete training and the fragmentation of training into several components. They also mentioned a number of factors that encouraged them to complete the training program, such as ease of use, the availability of continuing professional development credits, and the usefulness of content. We also found that training helped improve participants’ knowledge about risk communication and clinical knowledge.

A large proportion of the participants who committed to completing the training did not even access it. The literature suggests that relevance of the topic, quality of content and the provision of CPD credits are important incentive to participate in SDM training activities [12, 28]. These factors were likely not responsible for the observed limited access to the program, as we ensured that learners had a high interest in the content of the training program, and the training earned them CPD credits. Instead, the qualitative interviews suggest that a lack of time and logistical barriers caused the observed limited access to training. Cook et al. [28] similarly concluded that time required to complete the activity is an important determinant of learners’ selection of a CPD activity. Perhaps an online learning activity that would be broken into several smaller pieces would be preferable to the one-hour module we designed. Indeed, the *Pew Research Centre on Journalism and Media* examined 15 months’ worth of the most popular news videos on YouTube and concluded that the length for optimal engagement with online videos is between 2 and 5 min [29]. A modified version of the training program could therefore reflect these numbers, with modules as short as 2 min, to improve access.

Among the people who participated, a large proportion did not complete the training activities in their entirety, which agrees with several other reports of low retention in e-learning activities [30, 31]. We found the high dropout rates surprising, since we had tailored the training content and training component to the needs and preferences of learners [24], and our findings suggest that the learners found the training program useful and supportive of their clinical activities. High dropout rates may then be a consequence of the barriers participants mentioned in the interviews, such as lack of time, issues with Internet access, inconvenience of the training method, difficulty in using tools, or low peer and employer support. Similar barriers to physician engagement in self-directed e-learning in CPD were reported in a scoping review of 17 studies [32]. Resource requirements (including time, cost, and labour) and lack of information-technology skills were also reported as barriers to e-learning in health sciences education in a recent systematic review [33]. Additionally, research in education suggests that the learner’s isolation [34] and their inability to engage autonomously and actively in the learning process [35] might be important determinants of their participation in self-directed e-learning activities. We, however, did not assess these particular aspects in the current study. Because low completion rates of e-learning programs may undermine their effectiveness [30, 36], the factors influencing their participation should be considered and addressed by CPD developers to ensure the best possible learning outcomes.

We proposed several strategies to improve the learning modalities and implementation of the studied training program on SDM with older adults living with NCDs. The use of examples and role-plays are proven to be effective in training healthcare providers in SDM [37, 38]. Mamary et al. [39] recommend providing a user’s guide to learners who are less Internet-savvy. These authors also reported that computer training and dedicated time in the workplace for self-directed methods encouraged participation in self-directed continuing medical education. Some of the proposed strategies have been reported to support the participation of distance learners in CPD activities, such as providing access to a print version of the training material, lengthening the time available to complete training, offering individual profiles and follow-ups, proposing online collaboration, dividing modules into shorter sections, and supporting teamwork [40]. Another report suggests introducing a learning agreement between the learner and the university, offering support material, creating frequently asked questions (FAQs), using discussion boards, and monitoring learners’ opinions for continuous improvement [41]. Monetary incentives have also been demonstrated to influence HCPs participation to e-learning activities [42]. Podcasts could also be considered as this technology is becoming more and more popular for CPD training and information dissemination [43].

Even if this training program allowed improving some of participants’ knowledge, it did not allow increasing their intention to adopt SDM that was already high at baseline, nor their perceived capacity to adopt SDM. We also observed some improvement in clinical knowledge, but this is solely a secondary benefit of the training program that aimed at improving the adoption of SDM. Inclusion of outcomes at level of the older adults living with NCDs and their caregiver would have been required to conclude on the impact of this training program on patient care and quality of life from their own perspectives [44, 45]. However, we could not recruit enough older adults to assess whether training had actual impacts on the adoption of SDM. Studies with seniors with dementia consistently report high dropout rates both of seniors, caregivers and healthcare providers [46], and we were unsuccessful in addressing these challenges.

Trustworthiness of the findings is enhanced by our detailed description of the educational context and intervention, and by the use of multiple data sources (access data, surveys and interviews), methods (qualitative and quantitative), and researchers. Moreover, by collecting data both from the participants who completed training, and those who did not, we were able to provide an accurate picture of the factors at play. However, we did not ask feedback from participants on the qualitative data or interpretation of the data (member checking). We did not either use a control group in our quantitative evaluation of the impact of the training program on knowledge and intention, and so the results are prone to confounding bias since extraneous events or changes in context around the time of the intervention could have influenced the outcomes. The use of a non-random study sample and the high participant dropout rates may also have affected the results by introduction of selection bias. In addition, given the one-month delay between participation in the training activities and the interviews, recall bias is likely, and may have led to missing some important determinants to participation in the training program.

## Conclusions

Our study allowed us to identify important improvements for the development and implementation of this training program. In a next step, we plan to modify the program and implement it in a scaling-up experiment. The proposed list of strategies to counter the factors that hinder the participation of HCPs in interventions to improve SDM may be applied to several clinical contexts. These findings may support researchers in planning interventions targeting HCPs, especially those who practice in primary care contexts and those in the care of older adults living with NCDs. More studies that focus on actual SDM adoption following the implementation of professional training are now required.

## Data Availability

The datasets used and/or analysed during the current study are available from the corresponding author on reasonable request.

## Abbreviations

CPD: Continued Professional Development
DB: Decision Box
Min: Minutes
HCP: Healthcare provider
NCDs: Neurocognitive disorders
SDM: Shared Decision Making
